# Light/dark cycling causes delayed lipid accumulation and increased photoperiod-based biomass yield by altering metabolic flux in oleaginous *Chlamydomonas* sp.

**DOI:** 10.1186/s13068-019-1380-4

**Published:** 2019-02-21

**Authors:** Yuichi Kato, Yusuke Fujihara, Christopher J. Vavricka, Jo-Shu Chang, Tomohisa Hasunuma, Akihiko Kondo

**Affiliations:** 1 Engineering Biology Research Center, Kobe University, 1-1 Rokkodai, Nada-ku, Kobe, 657-8501 Japan; 2 Graduate School of Science, Technology and Innovation, Kobe University, 1-1 Rokkodai, Nada-ku, Kobe, 657-8501 Japan; 3 Department of Chemical Engineering, National Cheng Kung University, Tainan, 701 Taiwan; 4 Research Center for Energy Technology and Strategy, National Cheng Kung University, Tainan, 701 Taiwan; 5 Department of Chemical Science and Engineering, Graduate School of Engineering, Kobe University, 1-1 Rokkodai, Nada-ku, Kobe, 657-8501 Japan

**Keywords:** Biofuel, Biomass, *Chlamydomonas*, Light/dark cycling, Lipid, Metabolic profiling, Microalgae, Photoperiod, Carbohydrate

## Abstract

**Background:**

Light/dark cycling is an inevitable outdoor culture condition for microalgal biofuel production; however, the influence of this cycling on cellular lipid production has not been clearly established. The general aim of this study was to determine the influence of light/dark cycling on microalgal biomass production and lipid accumulation. To achieve this goal, specific causative mechanisms were investigated using a metabolomics approach. Laboratory scale photoautotrophic cultivations of the oleaginous green microalga *Chlamydomonas* sp. JSC4 were performed under continuous light (LL) and light/dark (LD) conditions.

**Results:**

Lipid accumulation and carbohydrate degradation were delayed under the LD condition compared with that under the LL condition. Metabolomic analysis revealed accumulation of phosphoenolpyruvate and decrease of glycerol 3-phosphate under the LD condition, suggesting that the imbalance of these metabolites is a source of delayed lipid accumulation. When accounting for light dosage, biomass yield under the LD condition was significantly higher than that under the LL condition. Dynamic metabolic profiling showed higher levels of lipid/carbohydrate anabolism (including production of 3-phosphoglycerate, fructose 6-phosphate, glucose 6-phosphate, phosphoenolpyruvate and acetyl-CoA) from CO_2_ under the LD condition, indicating higher CO_2_ fixation than that of the LL condition.

**Conclusions:**

Photoperiods define lipid accumulation and biomass production, and light/dark cycling was determined as a critical obstacle for lipid production in JSC4. Conversions of phosphoenolpyruvate to pyruvate and 3-phosphoglycerate to glycerol 3-phosphate are the candidate rate-limiting steps responsible for delayed lipid accumulation. The accumulation of substrates including ribulose 5-phosphate could be explained by the close relationship of increased biomass yield with enhanced CO_2_ fixation. The present study investigated the influence of light/dark cycling on lipid production by direct comparison with continuous illumination for the first time, and revealed underlying metabolic mechanisms and candidate metabolic rate-limiting steps during light/dark cycling. These findings suggest promising targets to metabolically engineer improved lipid production.

## Background

Biofuels are a sustainable alternative energy resource with potential to replace fossil fuels [1, 2]. Microalgae are promising biofuel producers due to rapid growth relative to land plants, and high availability of hydrosphere that can be utilized without competing with food production in farmlands [3, 4]. However, there are challenges for commercialization of biofuel production regarding processes of harvesting and lipid extraction [5]. Also, in the cultivation process, microalgae are required to achieve high lipid content together with high biomass production [6, 7]. Outdoor cultivation under photoautotrophic conditions using sunlight as an energy source and CO_2_ as a main carbon substrate is a cost-effective strategy for biofuel production. Periodic changes in light intensity throughout the day and night (hereafter, light/dark cycling) are an impactful factor for photosynthetic organisms including microalgae as these cycles are directly linked to biomass production under photoautotrophic conditions [8].

The influence of light/dark cycling on microalgal physiology has been studied mainly using the model green microalga *Chlamydomonas reinhardtii* (*C. reinhardtii*). Genome-wide gene expression analysis has revealed that many genes including those involved in cell cycle regulation and energy metabolism are expressed with strong diurnal periodicity [9]. Cell cycle progression is regulated by a circadian clock so that cell size increases during light periods and cell division occurs under dark periods [10, 11]. Cellular composition is also known to be affected by light/dark cycling. Carbohydrate content is affected by circadian rhythms, increasing during light periods, peaking in early dark periods and decreasing afterwards [12]. Unlike carbohydrate content, lipid content mainly depends on the state of cell division [13]. In addition, many central metabolites fluctuate throughout light and dark periods [14].

The influence of light/dark cycling on lipid accumulation and biomass production has been characterized in some microalgal species. In *Chlorella vulgaris*, higher biomass production and lipid content were achieved under 12 h:12 h and 18 h:6 h light:dark cycles relative to a 24 h:0 h continuous illumination condition [15]. In *Nannochloropsis* sp., lipid content and growth rate were highest under an 18 h:6 h light/dark cycle compared with 24 h:0 h and 12 h:12 h cycles [16]. In *Nannochloropsis gaditana*, biomass concentration was highest under a 12 h:12 h light/dark cycle, while lipid content was highest under a 16 h:8 h cycle, relative to 24 h:0 h and 8 h:16 h cycles [17]. In *Tribonema minus*, however, biomass production and lipid content were higher under 24 h:0 h continuous illumination compared with that under a more natural 12 h:12 h cycle [18]. In *Dunaliella viridis*, lipid content and cell division rates decreased under 12 h:12 h cycles compared with 24 h:24 h [19]. Despite these reports, the precise metabolic mechanisms underlying the influence of light/dark cycling on lipid accumulation and biomass production are still not well understood. Metabolic engineering using transgene and genome editing approaches have been reported as a useful tool for improving lipid productivity in some microalgae and cyanobacteria [20–22]. To fully demonstrate the potential of metabolic engineering techniques, knowledge of metabolic mechanisms, especially regarding rate-limiting steps, are necessary to select optimal target genes. Thus, lipid/biomass production and dynamic metabolic profiles must be determined while directly comparing light/dark cycling with continuous illumination to demonstrate the highest lipid productivity in outdoor conditions.

*Chlamydomonas* sp. JSC4 is a relative of the green microalga of *C. reinhardtii* isolated from the brackish water area in Taiwan [23, 24]. As a promising biofuel producer, JSC4 shows high growth potential and high lipid/carbohydrate content under salinity and photoautotrophic conditions. The metabolic mechanisms underlying lipid production in JSC4 have been revealed using comprehensive metabolome analysis and dynamic metabolic profiling by in vivo ^13^C labeling [25–28]. Cultivation of JSC4 under the optimal light intensity of 300 μmol m^−2^ s^−1^ resulted in higher lipid content and biomass production accompanied with higher levels of metabolites related to lipid synthesis (phosphoenolpyruvate; PEP, pyruvate, and acetyl-CoA; AcCoA) and starch synthesis/degradation (fructose 6-phosphate; F6P, glucose 6-phosphate; G6P, and glucose 1-phosphate; G1P) together with increased turnover of these metabolites [27]. In addition, JSC4 simultaneously achieved high biomass production (6407 mg L^−1^) and high lipid content (46.2%), which led to high lipid productivity (358.9 mg L^−1^ day^−1^) through activation of starch-to-lipid biosynthesis switching mechanisms under nitrogen depletion and salinity stress [28]. Under saline conditions, metabolic pool size and newly accumulated metabolites involved in lipid synthesis (pyruvate, AcCoA, and glycerol 3-phosphate; G3P) increased together with increased transcript levels of lipid synthesis-related genes (pyruvate decarboxylase, acetaldehyde dehydrogenase, and acetyl-CoA carboxylase) and starch degradation-related genes (starch phosphorylases), facilitating carbon metabolic flux switching from starch to lipid [28]. However, these previous results were obtained under continuous illumination, and the influence of light/dark cycling on lipid accumulation and biomass production of JSC4 has remained unreported.

In the present study, the effects of continuous light vs. light/dark conditions on lipid and biomass production in JSC4 were comprehensively compared for the first time. As a result, delayed lipid accumulation and increased biomass yield during light/dark cycling were revealed and the underlying metabolic mechanisms were elucidated. Metabolome analysis revealed that conversions of PEP to pyruvate and 3PGA to G3P are the candidate rate-limiting steps for lipid accumulation during light/dark cycling. Also, dynamic metabolic profiling suggested that enhanced CO_2_ fixation and increased biomass yield resulted from accumulating substrates including Ru5P during dark periods. These findings provide new insight into the improvement of microalgal lipid production during light/dark cycling, which is inevitable in outdoor cultivation.

## Results

### Alternations in biomass and lipid production under light/dark cycling and continuous illumination

**Fig. 1:**
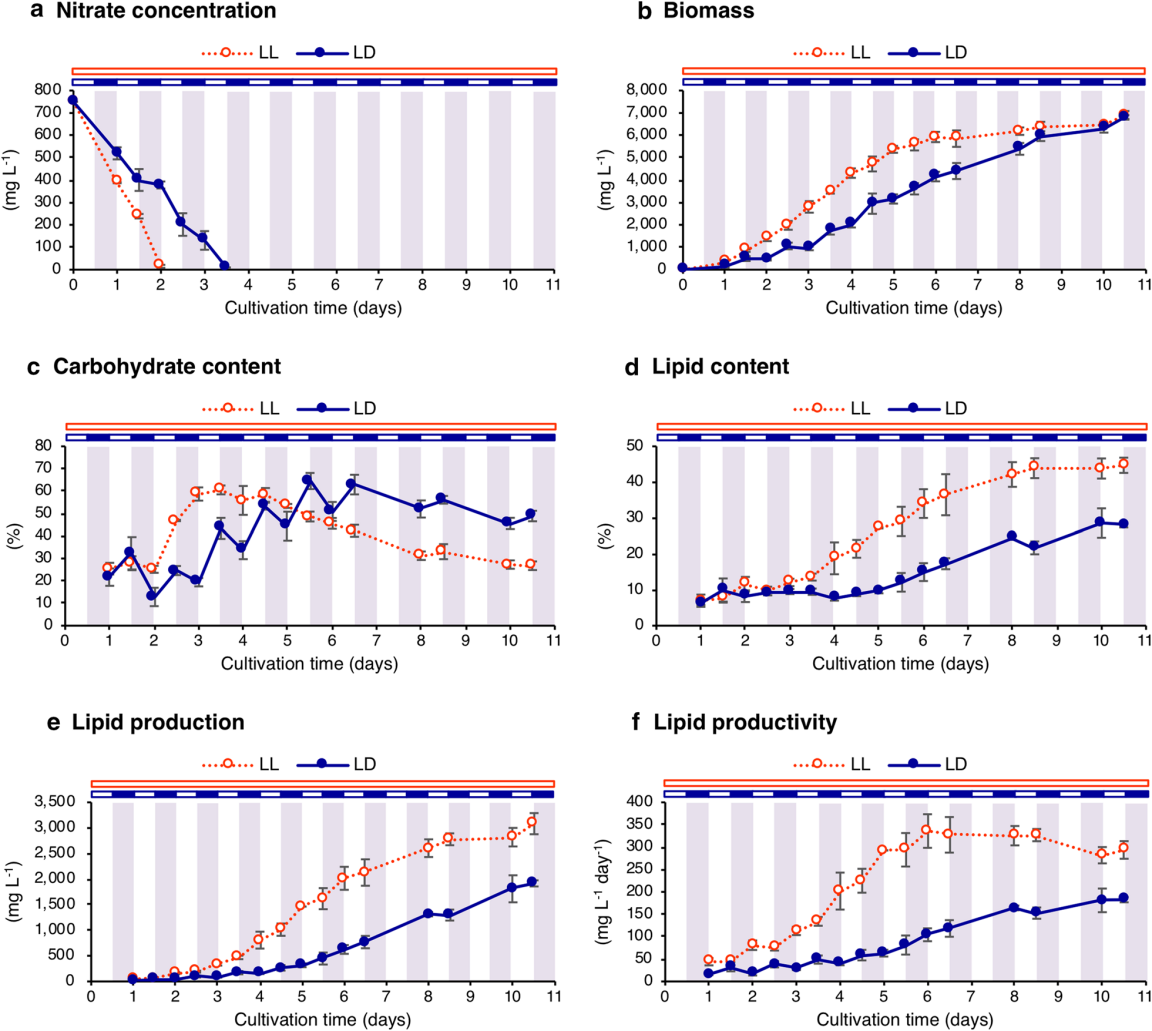
Time-course profiles of *Chlamydomonas* sp. JSC4 cultivated under the continuous light and light/dark conditions. Nitrate concentration (**a**), biomass (**b**), carbohydrate content (**c**), lipid content (**d**), lipid production (**e**), and lipid productivity (**f**) were analyzed under the continuous light (LL, red dotted line with open circle) and light/dark (LD, dark blue line with closed circle) conditions. Error bars indicate the standard deviation (SD) of three replicate experiments

To examine the influence of light/dark cycling on lipid accumulation and biomass production, JSC4 was cultivated under continuous light (LL) and light/dark (LD) conditions. Cultured cells were harvested every 12 h at dawn and dusk to discriminate the influences of light periods and dark periods on nitrate concentration, biomass production, carbohydrate content, and lipid content. Under LL conditions, the nitrogen source in the medium was completely consumed within 2 days (Fig. 1a). Under LD conditions, nitrogen consumption was relatively delayed with 3.5 days required for nitrogen depletion. Increase in biomass under the LL condition was observed during the initial 6 days, and a maximum biomass production of 6895.0 mg DCW L^−1^ was achieved at day 10.5 (Fig. 1b). Under LD conditions, biomass decreased during the dark period before nitrogen depletion, while it showed continuous increase after nitrogen depletion. A maximum biomass production of 6816.0 mg DCW L^−1^ under the LD condition was also achieved at day 10.5, and it was almost equal with that under the LL condition.

Time-course analyses of carbohydrate and lipid contents in JSC4 were carried out to examine lipid productivity and starch-to-lipid biosynthesis switching during light/dark cycling. Under LL conditions, carbohydrate content drastically increased immediately after nitrogen depletion at day 2.0 (Fig. 1c). A maximum carbohydrate content of 60.5% was achieved at day 3.5 when cells started accumulating lipid. Under the LD condition, carbohydrate content showed significant fluctuation throughout the experiment corresponding to the light condition. Carbohydrate content increased and decreased during the light periods and dark periods, respectively. Compared to LL conditions, accumulation and degradation of carbohydrate was delayed under the LD condition. The maximum carbohydrate content under LD conditions was 64.5% at day 5.5. Under LL conditions, lipid accumulation started at the day 3.5, which was 1.5 days after nitrogen depletion (Fig. 1d). Lipid content under LL conditions continued to increase after nitrogen depletion around day 8.5, and a maximum amount of 44.7% was achieved at day 10.5. Under LD conditions, lipid content started to increase at day 5.0, also 1.5 days after nitrogen depletion. Lipid accumulation was significantly delayed under the LD condition with a maximum lipid content of 28.6% at day 10.0.

Lipid production and productivity were calculated based on the results of biomass and lipid content (Fig. 1e, f). Under LL conditions, maximum lipid production was 3083.6 mg L^−1^, while it was 1916.0 mg L^−1^ under LD conditions. Lipid production under the LL condition became constant after day 8.5, which resulted from cessation of both biomass and lipid production. Under LD conditions, lipid production continued to increase at day 10.5. Maximum lipid productivity was 335.9 and 182.5 mg L^−1^ day^−1^ under LL and LD conditions, respectively. Lipid productivity under the LD condition continued to increase throughout 10.5 days cultivation, while that under the LL condition reached a maximum at day 6.0. Thus, lipid production was largely inhibited by LD conditions due to significant decreases in biomass production and lipid accumulation.

### Fluctuations in metabolic pool size under continuous illumination and light/dark cycling

**Fig. 2:**
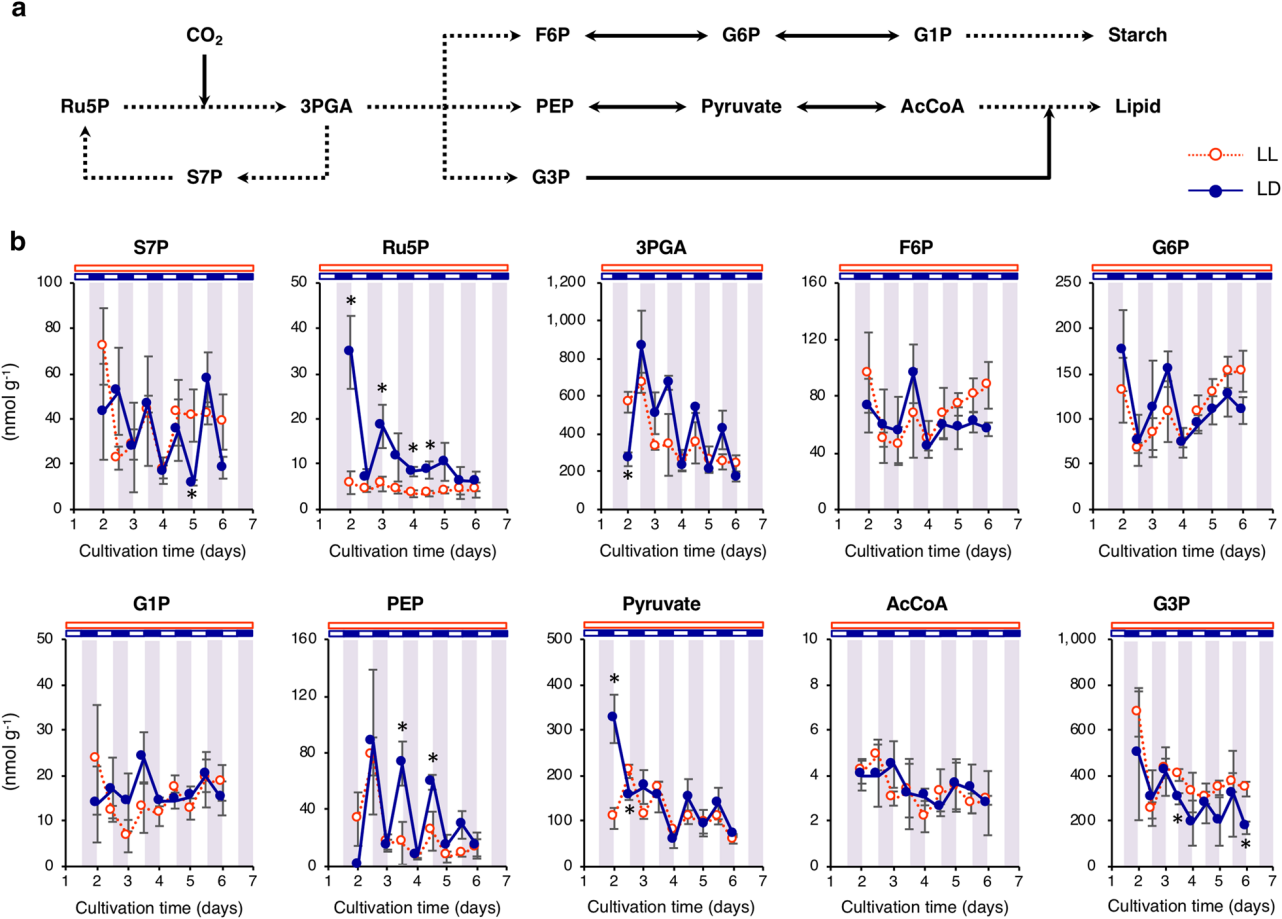
Profiles of the metabolic intermediates in starch and lipid synthesis. **a** Synthetic pathway of starch and lipid by CO_2_ fixation in *Chlamydomonas* spp. *S7P* sedoheptulose 7-phosphate, *Ru5P* ribulose 5-phosphate, *3PGA* 3-phosphoglycerate, *F6P* fructose 6-phosphate, *G6P* glucose 6-phosphate, *G1P* glucose 1-phosphate, *PEP* phosphoenolpyruvate, *AcCoA* acetyl-CoA *G3P*, glycerol 3-phosphate. Reactions containing multiple enzymatic steps are represented by dotted lines. **b** Pool size of the metabolic intermediates under the continuous light and light/dark conditions. Red dotted line with open circle and dark blue line with closed circle indicate the continuous light (LL) and light/dark (LD) conditions, respectively. Error bars indicate the SD of three replicate experiments (*p < 0.05 by Student’s *t* test)

Intracellular metabolites were comprehensively analyzed to elucidate mechanisms of delayed lipid accumulation under LD conditions. A simplified synthetic starch pathway, lipid production from CO_2_ and other key metabolites are shown in Fig. 2a. Among metabolites in the Calvin cycle, the level of sedoheptulose 7-phosphate (S7P) showed a diurnal periodicity under the LD condition that increased during light periods and decreased during dark periods (Fig. 2b). The level of ribulose 5-phosphate (Ru5P), another metabolite in the Calvin cycle, was drastically higher under LD conditions compared with LL conditions. In addition, Ru5P showed an opposite diurnal periodicity compared to that of S7P during the early stages of cultivation. Although carbohydrate content showed a distinct diurnal periodicity (Fig. 1c), the levels of F6P, G6P, and G1P did not show any diurnal periodicity with no difference between the two light conditions. In addition, there was no significant difference in the level of the key lipid intermediates pyruvate and AcCoA between the two light conditions. Similar to S7P, diurnal periodicity under LD conditions was observed for 3-phosphoglycerate (3PGA) and PEP, and the level of these metabolites at dusk was higher than that under LL conditions. The level of 3PGA dramatically increased during light periods, suggesting remarkable CO_2_ fixation under LD conditions. G3P, an important intermediate metabolite for lipid synthesis, also showed the same diurnal periodicity as that of S7P, 3PGA and PEP during the lipid accumulation stage. Under LD conditions, the level of G3P at dawn was lower than that of the LL condition.

### Biomass and lipid production evaluated on an exposed light basis

**Fig. 3:**
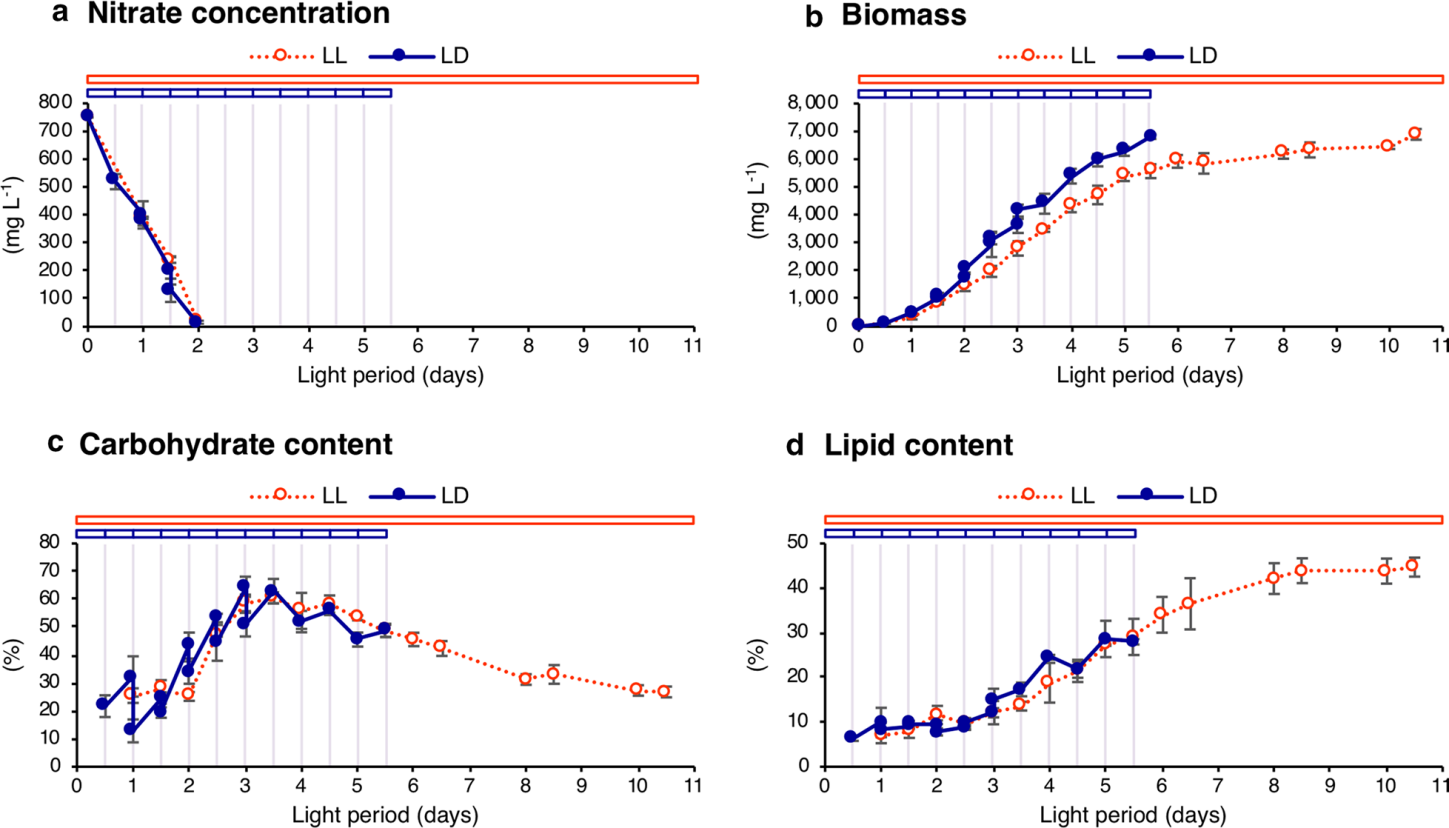
Time-course profiles of *Chlamydomonas* sp. JSC4 on an exposed light basis. Nitrate concentration (**a**), biomass (**b**), carbohydrate content (**c**), and lipid content (**d**) were analyzed under the continuous light (LL, red dotted line with open circle) and light/dark (LD, dark blue line with closed circle) conditions. Error bars indicate the standard deviation (SD) of three replicate experiments

Since light intensity was the same under both light conditions, exposed light energy per day under the LD condition was halved relative to that under the LL condition. This difference in exposed light energy between the two conditions could be a fundamental problem when directly comparing results of the LL and LD conditions. To examine the influence of this variable: exposed light energy, nitrate consumption, lipid/carbohydrate contents, and biomass production were compared by re-plotting the data of Fig. 1 under the LD condition to skip dark periods. When evaluated on an exposed light basis, it appeared that nitrogen was consumed at the same rate under LL and LD conditions, and nitrate was completely depleted after 2 days of light in both conditions (Fig. 3a). Furthermore, there was no significant difference in time-course changes of carbohydrate and lipid content between the two light conditions (Fig. 3c, d). Carbohydrate content under these light conditions started to increase after 1.5 light period days, reaching a maximum at day 3, followed by a gradual decrease. Under both light conditions, lipid content was around 10% during the first 3 light period days, followed by an increase to around 30% at 5.5 light period days. On the other hand, there was a significant difference in biomass yield under these light conditions with higher biomass yield under the LD condition relative to that of the LL condition (Fig. 3b).

### Dynamic metabolic profiles under continuous illumination and light/dark cycling

**Fig. 4:**
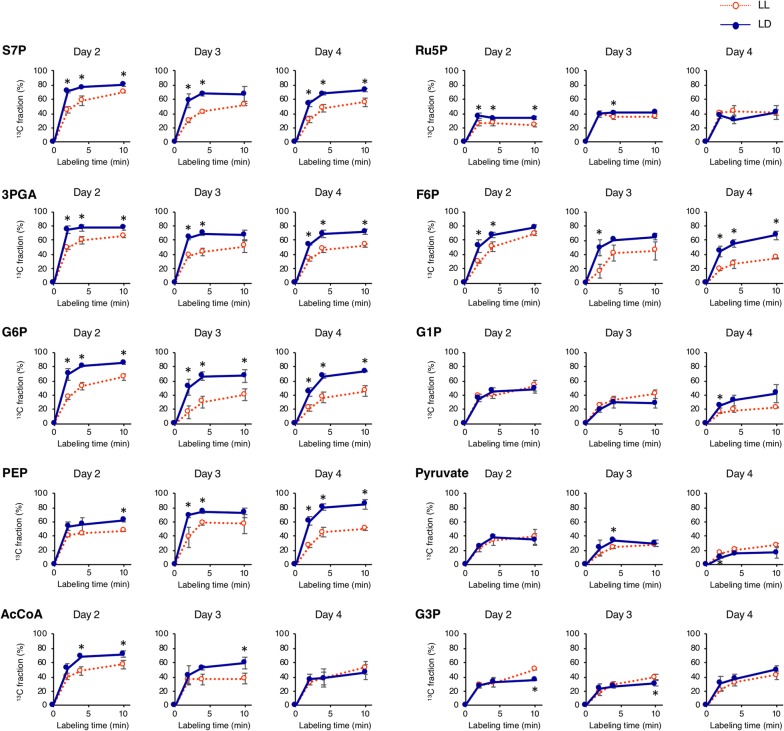
Time-course of ^13^C labeling under the continuous light and light/dark conditions. Red dotted line with open circle and dark blue line with closed circle indicate the continuous light (LL) and light/dark (LD) conditions, respectively. Error bars indicate the SD of three replicate experiments (*p < 0.05 by Student’s *t* test)

It was hypothesized that the increase in biomass yield under LD conditions was due to enhanced CO_2_ fixation during the light periods. To investigate the cause of this biomass increase and decreased lipid accumulation under LD conditions, metabolic carbon flux initiated from CO_2_ fixation was tracked using in vivo ^13^C labeling [28]. The ^13^C labeling experiments were conducted at days 2.0, 3.0, and 4.0 when cells actively fixed CO_2_ to accumulate biomass (Fig. 1). In each day, cultured cells were harvested and resuspended in medium containing NaH^13^CO_3_ to supply ^13^CO_2_ as a carbon source. Time-course sampling followed by metabolome analysis was performed to chronologically monitor carbon incorporation from CO_2_ into intracellular metabolites. The vertical and horizontal axes in Fig. 4 show ^13^C fraction in total carbon and labeling time after starting ^13^CO_2_ supply to the cells, respectively. ^13^C fraction indicates newly synthesized metabolites, and the inclination indicates turnover ratio of the metabolites.

Under LD conditions, the ^13^C labeling ratios of 3PGA and S7P were consistently and significantly higher throughout the experiment, while that of Ru5P, a downstream metabolite in Calvin cycle, showed only slight increase or no change compared with that of the LL condition. In the starch synthesis/degradation pathway, F6P and G6P, but not G1P, showed higher labeling ratios under LD conditions. In the lipid synthesis pathway, PEP showed an increased labeling ratio throughout the experiment; however, the labeling ratio of AcCoA only increased at days 2 and 3 under LD conditions. In addition, there was no remarkable change in the labeling ratio of pyruvate and G3P under LD conditions compared with that of the LL condition.

## Discussion

### Delayed progression of starch-to-lipid switching under light/dark cycling

Cultivation of microalgae using sunlight as the energy source is preferable for cost-effective biofuel production, although some outdoor conditions including light intensity are quite unstable. Light/dark cycling is one basic and inevitable condition of microalgal outdoor cultivation, and its influence on microalgal lipid production has been characterized in *C. reinhardtii* and in the other microalgal species [13, 15–19]. A recent study predicted growth and starch accumulation in *C. reinhardtii* under light and dark conditions using complex metabolic modelling as a powerful tool [29]. Despite these remarkable reports, the precise metabolic mechanisms underlying the influence of light/dark cycling on lipid production have not been fully understood. In addition, the influence of light/dark cycling on JSC4, which is a promising biofuel producer, must be elucidated to commercialize biofuel production using this strain. The present study investigated the influence of light/dark cycling on JSC4 biomass production and lipid accumulation by directly comparing continuous light and light/dark conditions. Time-course analysis of biomass concentration and carbohydrate/lipid contents revealed that biomass production and lipid accumulation significantly decrease under LD conditions (Fig. 1b, d). Compared with LL conditions, nitrogen depletion was also delayed 1.5 days under LD conditions (Fig. 1a). However, biomass production and lipid accumulation under the LD condition was delayed more than 1.5 days, suggesting that delay in nitrogen consumption is not the only negative influence of light/dark cycling. On the other hand, lipid accumulation started after 1.5 days after nitrogen depletion under both light conditions (Fig. 1a, d), suggesting that the activation of lipid synthesis is determined by nitrogen deficiency rather than light conditions.

Under LD conditions, JSC4 carbohydrate content peaked at dusk, which is consistent with the previous report in *C. reinhardtii* [12]. In *Nannochloropsis gaditana*, continuous increase in lipid content during light period has been reported [30], but this is only consistent with the early phase of JSC4 lipid production (Fig. 1d). In *Neochloris oleoabundans*, lipid content was not found to decrease from light/dark cycling while carbohydrate content decreased [31]. Thus, the influence of light/dark cycling on diurnal changes of cellular components appears to be different dependent on microalgal species.

The present study reveals for the first time that, when compared on an exposed light basis, transitions of lipid/carbohydrate content under LL conditions were similar with that under LD condition (Fig. 3c, d). This result suggests that the progression of starch-to-lipid switching mechanisms is positively affected by light illumination, and that lipid synthesis may be “delayed” but not “decreased” by light/dark cycling. Light exposure time should be a direct factor for lipid production, and there should be rate-limiting steps in lipid synthesis that are under the control of light illumination.

### Metabolic mechanism underlying delayed lipid accumulation during light/dark cycling

Metabolic pool size analysis and dynamic metabolic profiling under LD conditions were performed to elucidate metabolic mechanisms underlying delayed lipid accumulation. Under the LD condition, a significantly larger pool size of PEP was observed at the end of light periods compared to that of LL conditions (Fig. 2b). Increased levels of PEP should be derived from 3PGA, which also increased at the end of light periods. On the other hand, pyruvate fluctuations were different from that of PEP, and the level of ^13^C-labeled pyruvate did not remarkably increase under LD conditions while that of PEP increased significantly (Fig. 4). Together, these results suggest that conversion of PEP to pyruvate is a rate-limiting step of lipid synthesis during light/dark cycling. Similarly, the ^13^C fraction of G3P did not increase under LD conditions while that of 3PGA significantly increased (Fig. 4), suggesting 3PGA-to-G3P conversion as another rate-limiting step. Since decreased levels of G3P were detected by metabolic pool size analysis (Fig. 2b), lack of G3P might be a direct limitation of lipid synthesis under the LD condition. Thus, metabolic pool size analysis combined with dynamic metabolic profiling revealed the candidate rate-limiting steps in lipid synthesis during light/dark cycling. The genes responsible for the rate-limiting reactions would be the hopeful targets of metabolic engineering using tools recently developed in microalgae [20, 21].

In a previous study, conversion of pyruvate to AcCoA was identified as the metabolic rate-limiting step for lipid synthesis in JSC4 under continuous illumination conditions [28]. This difference in the determined rate-limiting steps suggests that these rate-limitations are culture condition-specific, and that the respective reactions may be more susceptible to negative influence from diurnal metabolic fluctuation caused by light/dark cycling. Similar results regarding dynamics of 3PGA and PEP during light/dark cycling were previously reported in *C. reinhardtii* [14]; however, diurnal fluctuation of these metabolites between dawn and dusk was more pronounced in JSC4 than in *C. reinhardtii*. This might be due to the higher potential of JSC4 to synthesize and convert these intermediate metabolites. Delayed lipid production during light/dark cycling is a critical problem for outdoor cultivation, and methods for accelerating lipid accumulation independent to light condition should be developed. Using a metabolomics approach, the present study identified candidate rate-limiting steps caused by light/dark cycling in the lipid synthesis pathway.

### Increased biomass yield from enhanced CO_2_ fixation during light period

Decreased biomass during dark periods was reported previously in green algae including *Chlorella sorokiniana*, *Nannochloropsis salina,* and *Picochlorum* sp. [32], suggesting an adverse influence of light/dark cycling on microalgal biomass yield. In the present study, intracellular carbohydrate was rapidly consumed during dark periods (Fig. 1c). However, a higher biomass yield under LD conditions was observed when considered on an exposed light basis (Fig. 3b). The level of ^13^C-labeled 3PGA, which is the direct product of CO_2_ fixation, increased under the LD condition (Fig. 4), suggesting higher CO_2_ fixation relative to that of the LL condition. Pool size analysis revealed that the level of Ru5P, which is upstream of 3PGA in the Calvin cycle, accumulated under LD conditions (Fig. 2b). Accumulation of Ru5P under LD conditions would be caused by the light-independent reactions in the Calvin cycle. Form these findings, it is hypothesized that the availability of abundant substrates might enhance CO_2_ fixation during light periods, resulting in increased biomass yields under LD conditions. Photo-independent activity during dark periods is another possible source of increased biomass yield under LD conditions on an exposed light basis [9], and this deserves further examination.

In a previous report, *Scenedesmus obliquus* and *Botryococcus braunii* produced higher biomass during 24 h:0 h continuous illumination, while *Neochloris* species’ biomass production was highest during 12 h:12 h light/dark cycling, even when factoring in cultivation time [8]. Similar observations were reported in *Neochloris oleoabundans* with higher biomass yield per mol photon under light/dark conditions relative to that of continuous light conditions [31]. When using LED illumination, maximum biomass production was observed under 24 h:0 h continuous illumination in *Nannochloropsis salina* and *Phaeodactylum tricornutum*, while that of *Isochrysis galbana* was observed under 18 h:6 h light/dark cycling [33]. In *Chlorella vulgaris*, higher biomass production under light/dark cycling was achieved in the order of 16 h:8 h, 12 h:12 h, 8 h:16 h [34]. Another *Chlorella vulgaris* study using blue LED and white fluorescent light reported that higher biomass production was achieved under light/dark cycling rather than continuous illumination [15]. Thus, influences of light/dark cycling on biomass yield also vary significantly depending on species.

As discussed above, cultivations of various microalgal strains were previously performed under light/dark conditions to evaluate influences on biomass, starch, and lipid production. However, the causative metabolic mechanisms underlying these influences remained to be established, along with understanding how light/dark cycling affects lipid accumulation and biomass production in the promising oleaginous microalga JSC4. The present approach revealed specific problems that could occur in outdoor cultivation of JSC4 together with the underlying metabolic mechanisms and candidate metabolic rate-limiting steps during light/dark cycling. The findings of the present study are essential for the commercialization of biofuel production using JSC4 and the other microalgae in the future.

## Conclusions

The current study verifies light/dark cycling as a critical obstacle of lipid production in JSC4. Metabolic pool size analysis combined with dynamic metabolic profiling revealed that conversions of PEP to pyruvate and 3PGA to G3P are the candidate rate-limiting steps during light/dark conditions. It is also noteworthy that CO_2_ fixation is enhanced during light/dark cycling, due to the accumulated substrates including Ru5P. Together these findings suggest promising targets for metabolically engineering improved lipid production.

## Methods

### Strain and culture conditions

The green microalga *Chlamydomonas* sp. JSC4 was isolated from a coastal area of southern Taiwan [23, 24]. The 18S rRNA sequence of JSC4 has been deposited in the National Center for Biotechnology Information GenBank with an accession number of KF383270. JSC4 was photoautotrophically cultured in double-deck photobioreactors, which is constructed by two flasks vertically joined, with a working volume of 70 mL (upper flask containing cell culture and lower flask containing 2 M NaHCO_3_/Na_2_CO_3_ to supply 2% CO_2_ gas) at 30 °C. The medium used was Modified Bold (MB) 6 N (8.82 mM NaNO_3_, 0.22 mM K_2_HPO_4_, 0.30 mM MgSO_4_, 0.17 mM CaCl_2_, 0.43 mM KH_2_PO_4_, 0.43 mM NaCl, and the metals described in the previous report) with 2% (w/v) sea salts (Sigma-Aldrich Co., St. Louis, MO, USA) [35, 36]. The culture was illuminated by white fluorescent lamps at light intensity of 250 μmol m^−2^ s^−1^ under the 24 h continuous light condition and the 12 h:12 h light/dark condition. Cells were pre-cultured for 3 days and inoculated at an initial cell density of 20 mg DCW L^−1^.

To determine residual nitrate concentration in culture medium, absorbance at 220 nm, where NaNO_3_ shows strong absorbance, was measured. Microalgal cultures were centrifuged at 5000×*g* for 1 min and the absorbance at 220 nm of the supernatant diluted 20-fold with distilled water was measured using a UV mini-1240 UV–Vis spectrophotometer (Shimadzu, Kyoto, Japan). The residual nitrate content was evaluated using a calibration curve [37].

To determine biomass concentration, cell cultures were harvested in microtubes, washed with distilled water once, and lyophilized overnight. DCW was determined by subtracting empty microtube weight from microtube weight with lyophilized cells. Dried cells prepared here were also used for the measurement of lipid and carbohydrate contents as described below.

### Measurement of lipid and carbohydrate contents

Lipid content of dried cells was measured as described previously [36, 38]. Briefly, the dried cells were fractured with 0.5 mm glass beads using a multi bead shocker (Yasui Kikai, Osaka, Japan), and then lipids were esterified using a Fatty Acid Methylation Kit (Nacalai Tesque, Kyoto, Japan). Identification and quantification of the fatty acid methyl esters (FAMEs) were performed by gas chromatography–mass spectrometry (GC–MS) using a GCMS-QP2010 Plus (Shimadzu) and a capillary column DB-23 (Agilent Technologies, Palo Alto, CA, USA). Heptadecanoic acid (Sigma-Aldrich Co.) was used as an internal standard.

Carbohydrate content of dried cells was measured using a hot acid hydrolysis procedure as described previously [28, 39]. Briefly, dried cells were suspended in 4% (v/v) H_2_SO_4_, autoclaved for 30 min at 120 °C, and then suspensions were neutralized by adding 22% (w/v) Na_2_CO_3_. After filtrating with a Shim-pack SPR-Pb column (Shimadzu), the glucose concentration in the supernatant was measured using HPLC (Shimadzu). The carbohydrate content in dried cells was quantified using soluble starch (CAS number: 9005-84-9, Nacalai Tesque) as a quantitative standard.

### Metabolome analysis

Sample preparation for metabolome analysis was performed as described previously with modifications [28]. Briefly, cultured cells were harvested using 1.0 μm pore size polytetrafluoroethylene filters (Merck Millipore, Burlington, MA, USA), washed with 20 mM ammonium carbonate (4 °C), and immediately suspended into 1 mL of methanol (− 30 °C) containing 33.4 μM piperazine-1,4-bis(2-ethanesulfonic acid) (Dojindo Laboratories, Kumamoto, Japan) as an internal standard. To prepare intracellular metabolites, 300 μL of chloroform (4 °C) and 100 μL of distilled water (4 °C) were added and the cells were fractured with 0.5 mm glass beads using a multi bead shocker (Yasui Kikai). After 325 μL of distilled water was added to 800 μL of the supernatant, the aqueous layer was filtered by a 3 kDa cutoff filter (Merck Millipore), dried, and re-dissolved in 20 μL of ultrapure water. Capillary electrophoresis–mass spectrometry (CE–MS) of intracellular metabolites was performed using G7100 CE and G6224AA LC/MSD time-of-flight systems (Agilent).

### In vivo ^13^C labeling

To evaluate the quantitative metabolic flux in the cells, in vivo ^13^C labeling was performed as described previously [28] using NaH^13^CO_3_ as a carbon source. Cultured cells were harvested and resuspended in MB6N containing 2% (w/v) sea salts and 25 mM NaH^13^CO_3_ (Cambridge Isotope Laboratories, Inc., Tewksbury, MA, USA) to the same cell density as that of the culture. Time-course labeling was performed for 2, 4, and 10 min at illumination of 250 μmol m^−2^ s^−1^ white fluorescent lamps. Intracellular metabolites were analyzed using CE–MS as described above. ^13^C fraction, which is the ratio of ^13^C in total carbon, was calculated for each metabolite by detecting mass shifts from the ^12^C to ^13^C mass spectra.

## Abbreviations

AcCoA: Acetyl-CoA
DCW: Dried cell weight
F6P: Fructose 6-phosphate
G1P: Glucose 1-phosphate
G6P: Glucose 6-phosphate
G3P: Glycerol 3-phosphate
LD: Light/dark
LL: Continuous light
PEP: Phosphoenolpyruvate
3PGA: 3-Phosphoglycerate
Ru5P: Ribulose 5-phosphate
S7P: Sedoheptulose 7-phosphate
SD: Standard deviation
